# Profile and primary treatment outcomes in membranous nephropathy

**DOI:** 10.15537/smj.2022.43.9.20220459

**Published:** 2022-09

**Authors:** M.K. Hemanth Kumar, Jashan Sandhu, Jasvinder S. Sandhu

**Affiliations:** *From the Department of Nephrology (Kumar, J. S. Sandhu); from the Department of Pathology (J. Sandhu), Dayanand Medical College and Hospital, from the Department of Pathology (J. Sandhu), Gian Sagar Medical College and Hospital, and from the Department of Nephrology (J. S. Sandhu), Satguru Partap Singh Hospital, Punjab, India.*

**Keywords:** idiopathic membranous nephropathy, nephrotic syndrome, cyclophosphamide, tacrolimus, rituximab

## Abstract

**Objectives:**

To assess the clinical profile and primary treatment response and outcomes in idiopathic membranous nephropathy (IMN) patients.

**Methods:**

This study was carried out between December 2013 and January 2019 in a tertiary care hospital in North India on 2 years retrospective and 3 years prospective renal biopsy proven patients with IMN presenting with nephrotic syndrome. Basic baseline investigations carried out were urinary proteins, serum albumin, serum creatinine, other special tests wherever necessary or possible (including phospholipase A2 receptor antibodies), and different treatment regimens were offered for treatment. The patients were followed up for a minimum period of 6 months after administration of treatment.

**Results:**

The study was carried out in 120 patients with mean age of 43±14.6 years and male female ratio of 1.65:1. Hypertension was noted in 36%, microscopic hematuria in 13%, and mean 24 hours urinary proteinuria 10.5±3.1 gm. Complete or partial response at 6 months was observed in 57% and 34% cases to cyclophosphamide, 60% and 40% to modified Ponticelli treatment, 81% and 19% to tacrolimus, and 40% and 36% cases to rituximab. Relapse was observed in 6% of cyclophosphamide and 13% in tacrolimus groups.

**Conclusion:**

Our results show a good and comparable response to cyclophosphamide, tacrolimus, and rituximab at 6 months of follow up. The cases which achieved complete remission had significantly lower baseline proteinuria compared to those who did not respond.

Membranous nephropathy (MN)is an autoimmune disorder in which pododcytic antigens in glomeruli are targeted by circulating auto-antibodies.^1^ It is the leading cause of nephrotic syndrome in adults.^2^ Most of adults (80%) with idiopathic membranous nephropathy (IMN) present with nephrotic syndrome and the rest present with sub nephrotic proteinuria. Spontaneous remissions and relapses are seen in the disease course. Approximately 20% of the patients may show spontaneous complete remission and another 15-20% undergo partial remission.^3^

Circulating autoantibodies to phospholipase A2 receptor (PLA2R) are present in 70-80% of cases. Immunosuppressive therapy is recommended for patients with persistent nephrotic syndrome. Various treatment regimens have been used for IMN including methylprednisolone, cyclosporine, chlorambucil, cyclophosphamide, tacrolimus, and recently rituximab.^2^

It has been seen that corticosteroids and alkylating agents used in combination were helpful in maintaining and preserving long term kidney function. Recently, rituximab has been a major player as it avoids long term nephrotoxicity associated with alkylating agents and calcineurine inhibitors and also avoids the high risk of relapse seen with them.^3^ However, rituximab therapy is still relatively more expensive than other therapies, and clinicians often have to use a multi-drug approach keeping the side effects, rate of remission, and cost factors to patient in mind. The aim of this study is to determine the outcomes of IMN.

## Methods

This study was carried out in the Department of Nephrology, Dayanand Medical College and Hospital, Punjab, India. The study was approved by the Institutional Ethics Committee on Human Research and was carried out in accordance to the principles of Declaration of Helsinki.

The cases included 2 years of retrospective and 3 years prospective renal biopsy proven patients with IMN presenting with nephrotic syndrome who were on regular follow up in the Nephrology Outpatient Department between December 2013 and January 2019. Only patients with a minimum follow up period of 6 months were included in the study. Patients with secondary causes of nephrotic syndrome were excluded. Wherever possible, patients were followed up for a year.

After obtaining written informed consent, a detailed history was obtained with regard to the chief complaints, duration, past medical history, family history, and drug history. On subsequent visits, a history was obtained to identify any possible complications arising from treatment or disease. All patients underwent a systematic physical examination. All the patients were subjected to basic investigations including hemogram, blood urea, serum creatinine, urine routine examination, 24-hour urine proteins or spot urine protein-creatinine ratio, serum albumin, serum proteins, serum cholesterol, blood sugar, and serum electrolytes. In the retrospective group, patients with biopsy proven primary nephrotic syndrome patients were studied. In the prospective group all patients presenting with nephrotic syndrome underwent ultrasound-guided kidney biopsy and the specimen was examined under light microscopy and immunofluorescence. Whenever indicated, special investigations were carried out like complement levels C3 and C4, hepatitis-B Ag, anti-hepatitis C virus, human immunodeficiency virus, and anti-nuclear antibodies. Wherever possible, PLA2R levels were also measured at baseline. The patients were treated on the established line of treatment in the hospital at the discretion of the treating nephrologist.

Different treatment regimens offered to the patients as first line of therapy included oral cyclophosphamide (2 mg/kg) administered for either 12 or 16 weeks plus oral prednisolone (1 mg/kg) with tapering was used as the initial treatment regimen in 50 patients.

The modified Ponticelli regimen was used in 5 cases who were administered pulse intravenous methylprednisolone 1 g/day for 3 days followed by oral prednisolone (0.5 mg/kg/day) for 27 days in months 1, 3, and 5 and this was alternated with oral cyclophosphamide (2 mg/kg) daily in months 2, 4, and 6.^4,5^

Oral tacrolimus 0.05-0.1 mg/kg (adjusted for trough serum tacrolimus levels of 6-8 micrograms/L) for 6-12 months plus oral prednisolone 1 mg/kg/day for 3 months with tapering was used as the initial treatment regimen in 17 cases.

A total of 50 patients were treated with rituximab as initial line of therapy. They were administered 4 weekly doses of 375 mg/m^2^ rituximab intravenously or 1000 mg of rituximab on day one and 15 in the last 4 patients. A single booster dose of 375 mg/m^2^ rituximab was administered at 6 months in 4 patients.

The following criteria were used to define: i) complete remission; ii) reduction of proteinuria to <0.30 g/day or urine PCR<0.3 or trace or negative results on repeat dipstick; iii) partial remission: reduction of proteinuria to between 0.31-3.4 g/day or a decrease in proteinuria of ≥50% from baseline; iv) time to remission: the time from initiation of therapy to the first day after which remission was observed; v) relapse: proteinuria ≥3.5 g/day or urine dipstick ≥3+ occurring after complete remission for >1 month.

### Statistical analysis

All statistical analyses were carried out by the Statistical Package for the Social Sciences, version 17.0 (SPSS Inc., Chicago, IL, USA). Statistical analysis was carried out using nonparametric exact statistical methods such as the Fischer exact test for categorical variables, Mann-Whitney-U test for continuous variables between 2 groups, and Kruskal-Wallis test for continuous variables among 3 or more groups. Values of continuous variables are reported as the mean ± standard deviation (SD) where appropriate. A *p*-value of <0.05 was considered significant.

## Results

The total number of patients who presented with nephrotic syndrome and were diagnosed with IMN was 122 (76 males, 46 females). The mean age of the patients with IMN was 43±14.6 years and the highest proportion of patients were in the 3^rd^-4^th^ decade constituting 52.4% of cases with MN, followed by patients in the 5^th^-6^th^ decade accounting for 39.3% of cases. The male to female ratio was 1.65:1. Hypertension was observed in 36% patients with MN with the highest incidence in patients aged >40 years. The incidence of microscopic hematuria was 13% in the study group. The baseline characteristics of cases with MN- baseline proteinuria was 10.5±3.1 g/day, serum albumin was 2.4±0.4 g/dL, and serum creatinine was 1.04±0.58 mg/dL. There was a progressive increase in mean baseline proteinuria with increasing age. Protein excretion was relatively higher among patients >60 years of age (11.5±2.08 g/g) than other age groups, but the difference was insignificant (*p*=0.725). The mean serum creatinine was relatively higher in the 4^th^-5^th^ decade as compared to other age groups. Considering an arbitrary value of serum creatinine of 1.2 mg/dL to define renal dysfunction, the incidence of patients with or without renal dysfunction was defined. In our study renal dysfunction was observed in 14% (17 cases) of patients with MN (Table 1).

**Table 1:** - Baseline charcteristics (N=122).

Clinical parameters	n (%)
Age, years (mean±SD)	43±14.6
Male	76 (62.3)
Female	46 (37.7)
Hypertension	44 (36.0)
Microscopic hematuria	16 (13.0)
Renal dysfunction	17 (14.0)
Serum creatinine, mg/dl (mean±SD)	1.04±0.58
Serum albumin, g/dl (mean±SD)	2.4±0.4
Proteinuria, g/24 hours (mean±SD)	10.5±3.1
Anti-PLA2R positive	24/37 (64.8)
Anti-PLA2R titre, RU/ml (mean±SD)	166.9±244.25

Values are presented as number and percentage (%), mean ± standard deviation (SD). PLA2R: phospholipase A2 receptor, mg/dl: milligrams per deciliter, g/dl: grams per deciliter, RU/ml: relative units per milliliter

We observed that the patients who achieved a primary remission in MN were relatively older than those who achieved only partial remission or no response to primary treatment. However this difference was not statistically significant (*p*=0.736). Hence, age did not significantly affect the treatment response.

Patients who achieved complete remission to the primary treatment regimen had significantly lower baseline proteinuria than those who did not respond (*p*=0.013). Older patients took relatively longer time to complete remission as compared to younger patients with IMN. However the difference was not statistically significant (*p*=0.512).

Patients with higher degree of proteinuria took longer time to complete remission but the difference was not statistically significant (*p*=0.661). We did not observe any significant difference in the baseline proteinuria between relapsers and non-relapsers among those patients who had achieved a complete remission (*p*=0.44).

We did not observe any significant age difference between relapsers and non-relapsers among those patients who had achieved a complete remission (*p*=0.844). We observed that relapsers took longer time to achieve primary complete remission as compared to non-relapsers, but the difference was not statistically significant (*p*=0.150).

Two patients died of sepsis (one treated with cyclophosphamide and one with tacrolimus) and were excluded from further analysis. A total of 55 patients received oral cyclophosphamide of which 5 received modified Ponticelli regimen. The rates of complete and partial remission with oral prednisolone plus oral cyclophosphamide were 57% and 34.7% and those with modified Ponticelli regimen were 60% and 40% (Table 2).

**Table 2:** - Primary treatment outcomes.

Primary response	Prednisolone, cyclophosphamide (n=49)	Modified Ponticelli (n=5)	Prednisolone, tacrolimus (n=16)	Rituximab (n=50)
Males	34 (69.4)	3 (60.0)	9 (56.3)	28 (56.0)
Females	15 (30.6)	2 (40.0)	7 (43.7)	22 (44.0)
Complete remission	28 (57.0)	3 (60.0)	13 (81.0)	20 (40.0)
Partial remission	17 (34.7)	2 (40.0)	3 (19.0)	18 (36.0)
No response	4 (8.0)	0 (0.0)	0 (0.0)	12 (24.0)
Relapse	3 (6.0)	0 (0.0)	2 (13.0)	1 (2.0)

Values are presented as number and percentage (%).

The time to remission with oral prednisolone plus oral cyclophosphamide was 7.7±2.1 weeks and that with modified Ponticelli regimen was 10.66±2.31 weeks. The cases that achieved a complete remission on oral cyclophosphamide plus oral prednisolone had a significantly lower baseline proteinuria (9.4±2.9 g/24 hours) as compared to those who did not have any response (12.9±2.6 g/24 hours). Patients with massive proteinuria (>10 g/24 hours) had a longer time to complete remission (10.4±4.7 weeks) as compared to those with <6 g/24 hours proteinuria (8.8±4.4 weeks).

The relapse rate in our study with oral cyclophosphamide was 6% and the time to relapse was 9±3 weeks. Oral tacrolimus plus oral steroids in appropriate doses adjusted for trough tacrolimus levels were used both as a first and second line treatment in cases with MN. A total of 17 cases received oral tacrolimus plus oral prednisolone as the initial treatment regimen. The remaining patients received tacrolimus after relapse or cyclophosphamide resistance. In our study, the rate of complete remission with tacrolimus was 81%. In our patients who received tacrolimus, those who achieved only partial remission were relatively older age (44 years) than those who achieved complete remission (28 years), although the difference was not statistically significant. We also observed that those patients who had only a partial response to oral tacrolimus had relatively higher degrees of proteinuria (11 g/day) than those who had complete remission (10 g/day), although the difference was not statistically significant. The time to complete remission in our study was 13 weeks after the administration of oral tacrolimus. The relapse rate after oral tacrolimus administration was 13%, and the mean time to relapse was 4 weeks after treatment completion.

A total of 50 patients were treated with rituximab as initial line of therapy. Baseline characteristics included serum creatinine (1.06±0.18 mg/dL), urine proteinuria (9.58±6.3 gm/24 hours), and serum albumin (2.5±0.6 g/dL). Baseline PLA2R levels were available for 37 patients (166±244 RU/mL). Follow-up levels of PLA2R were only available in few cases and a reduction in levels were observed after the completion of therapy.

Mean 24-hour urinary proteins at baseline was less in patients achieving complete remission (6.0±3.4 g/24 hours) and partial remission (8.2±2.6 g/24 hours), compared to those who did not show remission (12.1±2.2 g/24 hours). However, this difference was not statistically significant.

The median time to complete remission was 6.3 months. Relapse was observed in only one (2%) case at the 7^th^ months. The rate of complete remission with rituximab was 40%, and partial remission was observed in 36% of the cases at 6 months. Approximately 24% cases failed to achieve remission after 6 months of therapy initiation.

Seven patients developed diabetes mellitus (all of which cases had received tacrolimus), 8 developed pneumonia, of which 2 had life threatening sepsis (6 cases had received tacrolimus and 2 received cyclophosphamide), 7 cases developed urinary tract infection, 6 developed neutropenia (all were exposed to cyclophosphamide), 3 had venous thrombosis (2 deep vein thrombosis of lower limbs and one renal vein thrombosis), and one developed seizures. No adverse effects were observed with rituximab treatment.

## Discussion

Other authors have observed a lower degree of proteinuria in studies from Pakistan, Europe as well as North India with proteinuria of 7.2 g/24 hours, 7.1 g/ 24 hours, and 6 g/24 hours respectively.^5-7^ A similar incidence of hypertension and microscopic hematuria was observed in some studies,whereas others showed a higher incidence of hypertension.^5,6^ A total of 50 patients received oral cyclophosphamide plus oral steroids as first line treatment in our cases with IMN and 5 received a modified Ponticelli regimen. A complete response was observed in 57% of patients on cyclophosphamide and 60% of patients on modified Ponticelli regimen. The time to remission was 7.7±2.1 weeks for cyclophosphamide and 10.66±2.31 weeks for modified Ponticelli regimen. The relapse rate was 6%.

Two studies from India by Jha et al^5^ and Ram et al^8^ have reported lower remission rates. Jha V et al^5^ reported a complete remission rate of 32% and partial remission rate of 40%. Ram et al^8^ reported a complete remission rate of 31% and partial remission rate of 46.5% at 9 months.

A relapse rate of 6.7% with oral cyclophosphamide was reported by Ramachandran et al^9^ from India which was similar to our study (6%). Other studies from Japan by Eriguchi et al^10^ reported higher relapse rates of 30% and Jha V et al^5^ from India reported higher relapse rates of 23%. Our rates of complete remission with tacrolimus was 81%, which was similar to that reported studies by Cui et al^11^ which showed remission rates of 81% and Ramachandran et al^9^ which showed remission rates of 74%. Some other older studies showed lower remission rates. A study on 408 cases by Qin et al^12^ observed that the patients who achieved remission were relatively younger than those who did not have a response, which was similar to our findings with tacrolimus. The time to remission with tacrolimus in our study was 13 weeks which was similar to that noted by Liang et al^13^ (3.2 months). Our rates of relapse with tacrolimus (13%) were comparable to those observed by Cui et al^10^ (16%) and Liang et al^13^ (10%), whereas the study by Ramachandran et al^8^ reported a relapse rate of 40% and Qin et al^11^ reported a relapse rate of 37%.

Rituximab causes selective depletion of B cells which helps achieve long-term complete or partial remission in these patients. Rituximab was administered to 50 patients with IMN. We also studied the levels of PLA2R antibodies in selected patients who could afford the cost of the test. In patients receiving rituximab, the median time to remission was 6.3 months with complete remission rate of 40% and partial remission rate 36% at 6 months post therapy. Only a small number of patients were successfully followed up for one year and thus were not statistically analyzed; however it was observed that remission rates improved with time. Ruggenenti et al^14^ reported in their study that rituximab induces nephrotic syndrome remission in 2/3^rd^ patients with IMN. This was also similar to a study by Teisseyre et al^15^ which documented clinical remission (complete and partial) in 53% cases at 6 months and 60% cases at 12 months.

Cattran et al^16^ in a study on quantifying remission duration on outcome, validated and quantified partial remission and complete remission as surrogates for long-term outcome in MN. Dahan K et al^17^ in a randomized trial at 31 French hospitals for severe MN in a 6-month trial with follow-up showed that serum albumin and PLA2R-antibodies levels are early markers of non-suppressive antiproteinuric treatment-rituximab efficacy, whereas the effect on proteinuria and the remission appeared after 6 months.

Ou et al^18^ evaluated the efficacy of rituximab for MN in a systematic review and meta-analysis of 11 studies in a total of 723 participants and observed that rituximab significantly improved cumulative remission (*p*=0.007) compared with other treatments such as cyclosporine, cyclophosphamide, and steroids. They concluded that rituximab could improve the rate of clinical remission and was more effective than other treatments in reducing relapse rates.

### Study limitations

While we were able to compare the short-term efficacy of various drugs used, the follow-up period in our cases treated with rituximab was insufficient to document and compare its long-term efficacy with that of the other drugs. Another limitation of this study was that a detailed histopathological evaluation of the renal biopsies was not included as a part of our original study protocol. A detailed assessment of the degree of glomerular involvement, interstitial fibrosis, as well as co-existing vascular lesions in renal biopsies may yield more accurate clinicopathological correlation of cases and this needs to be studied further in future trials.

Baseline proteinuria in IMN is an important parameter to assess response to therapy as it is significantly higher in patients with no response than those with a complete remission. The mean time to complete remission is also relatively longer in cases with massive proteinuria (>10 g/24 hours) and in older patients. The patient demographics play an important role in deciding the primary treatment options for different patients as several parameters need to be given due consideration in making this decision, including time to remission, relapse rate, complications like opportunistic infections, drug toxicity, as well as cost of therapy.

In conclusion, this study was carried out in a large cohort of patients with IMN, showed favorable response to existing therapies with cyclophosphamide, tacrolimus, and rituximab. The time to relapse was significantly shorter following oral administration of tacrolimus than with cyclophosphamide and rituximab. Unlike alkylating drugs, rituximab takes a longer time to achieve complete and partial remission.
